# The Treatment of Symptomatic Diffuse Proliferative Cerebral Angiopathy With Cilostazol: A Case Report

**DOI:** 10.7759/cureus.63387

**Published:** 2024-06-28

**Authors:** Diwas Gautam, Daryl E Morrison, Michael T Bounajem, Lubdha M Shah, Ramesh Grandhi

**Affiliations:** 1 Neurosurgery, University of Utah, Salt Lake City, USA; 2 Radiology, University of Utah, Salt Lake City, USA

**Keywords:** vascular pathology, arteriovenous malformation (avm), computed tomographic angiography, steal phenomenon, aphasia, cilostazol, diffuse proliferative cerebral angiopathy

## Abstract

Diffuse proliferative cerebral angiopathy (DPCA) is a rare form of cerebral vascular malformation responsible for 3.4% of all cerebral arteriovenous malformations (AVMs). The relative risk of rupture for DPCA is lower than for classic AVMs, so they are often managed medically. Despite the somewhat lower rupture risk, the risk of rebleeding is paradoxically higher than in classical AVMs, and there is a potential for significant morbidity and mortality. The aim of this article is to describe a case of a patient with symptomatic DPCA who experienced symptomatic improvement after treatment with the vasodilating agent cilostazol. This is a case report of a patient who presented with aphasia and was found to have DPCA. CT perfusion with acetazolamide challenge confirmed that the patient’s symptoms were due to steal phenomena. Subsequently, the patient was treated with 50 mg of cilostazol daily to mediate a vasodilatory response within the arterial tree. Within three days of treatment with cilostazol, the patient showed significant improvement in his aphasia. The patient returned to the clinic a month later and reported continued improvement in his aphasia with speech therapy. Patients who present with neurological deficits from steal phenomena caused by DPCA are difficult to treat surgically because DPCAs often involve normal parenchyma. We present an example of a noninterventional alternative, oral cilostazol paired with functional rehabilitation, for alleviating symptoms associated with DPCA.

## Introduction

Diffuse proliferative cerebral angiopathy (DPCA) is a form of cerebral vascular malformation responsible for 3.4% of all cerebral arteriovenous malformations (AVMs) [1]. Defined by the lack of a dominant feeder artery to a large nidus, proximal artery stenosis, diffuse angiogenesis, and interspersed normal brain tissue within the malformation [1], DCPAs are an uncommon vascular pathology that is phenotypically distinct from general AVMs. Whereas classic AVMs are regularly managed with direct intervention after risk assessment, DPCAs are typically managed conservatively because surgical intervention carries a heightened risk for severe neurological deficit because of the significant entanglement of normal brain tissue within nidus [2, 3]. The relative risk of hemorrhage from DCPA is lower than that of classical AVMs, although it is not uncommon for patients to experience a gradual decline in executive function as a result of repeated minor bleeding, and death can still result from major rupture [4, 5]. This case details the successful use of cilostazol for the management of symptoms in a patient who presented with aphasia and was found to have DCPA. The observed improvements in our patient's symptoms are, to our knowledge, the first of their kind to be reported after vasodilation therapy with cilostazol.

## Case presentation

A 45-year-old man with no pertinent past medical history was transferred to our institution with two days of antecedent nausea and vomiting followed by one day of worsening headaches and difficulty with speech production and comprehension. His neurological examination was notable for an aphasia picture with difficulties in understanding commands and speech production. He did not exhibit any other focal neurological deficits.

**Figure 1:**
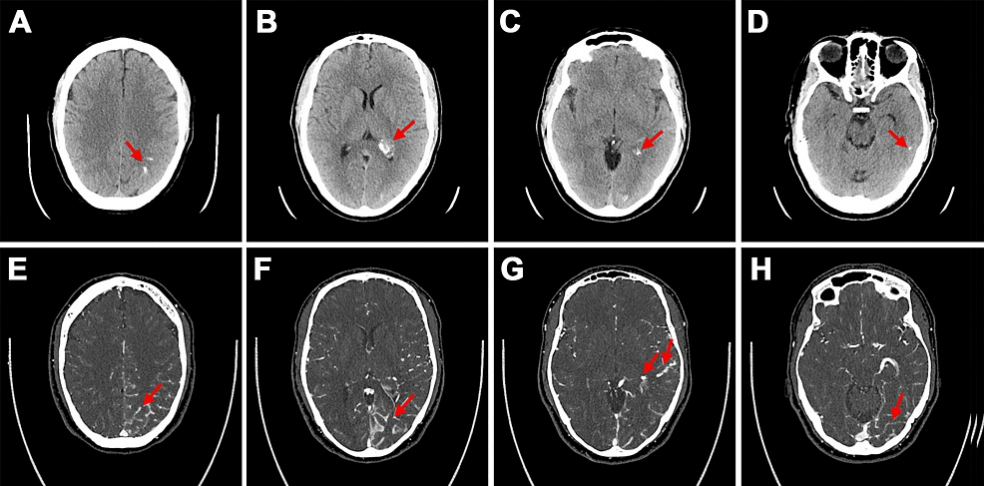
Diagnostic imaging Initial non-contrast head CTs (A-D) show curvilinear parenchymal calcification in the left parietal, occipital, and temporal lobes and asymmetric prominence and calcification of the left atrial choroid plexus (red arrows). Head CTA images (E-H) show prominent vascular structures insinuating along the left parietal, occipital, and temporal lobes consistent with a diagnosis of diffuse proliferative cerebral angiopathy (red arrows).

**Figure 2:**
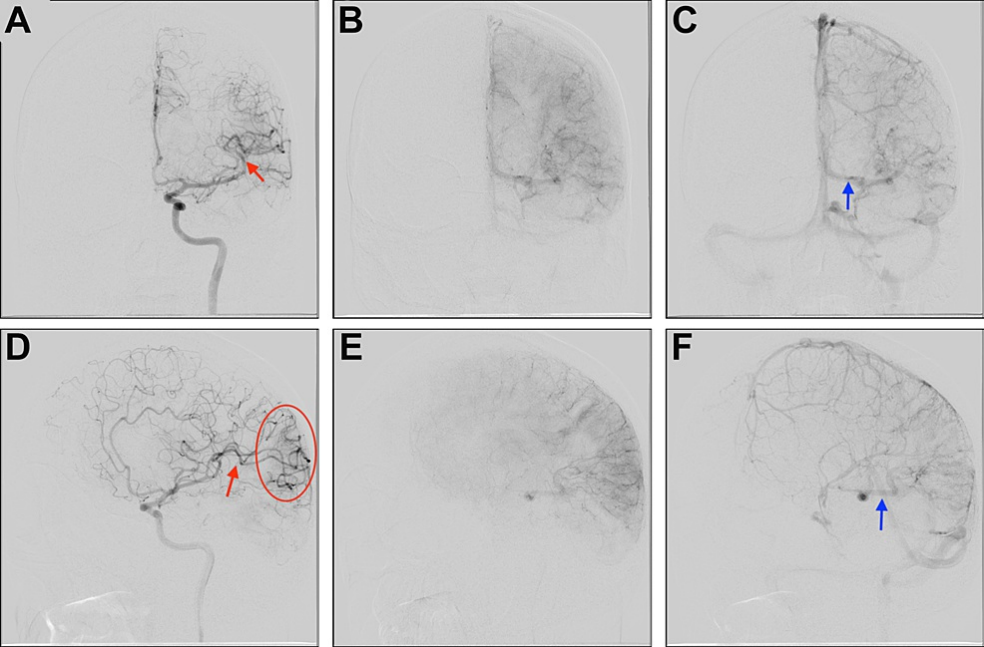
Diagnostic cerebral angiogram (A-F) Images demonstrate diffuse proliferative cerebral angiopathy (DPCA) with feeders from the left middle cerebral artery (red arrows) and early drainage into a dilated left-sided basal vein of Rosenthal (blue arrows). The red oval indicates the subtle pathology with early capillary blush in the left parieto-occipital area.

A head computed tomography (CT) scan performed before arrival at our institution revealed curvilinear hyperattenuating lesions in the left parietal, temporal, and occipital lobes with regional mass effect that raised concern for possible hemorrhage related to intra-axial mass or AVM (Figure 1). Subsequent head CT angiography (CTA) at our institution demonstrated prominent vascular structures insinuating along the sulci of the left parietal and temporal lobes, which was suspicious for a ~2 cm vascular malformation. The prominence of the left middle cerebral artery branches and the asymmetrically dilated left basal vein of Rosenthal suggested arterial feeders and venous drainage, respectively. No vascular tuft was identified to suggest a discrete nidus. Similarly, brain magnetic resonance imaging (MRI) demonstrated prominent vascular structures along the left occipital, temporal, and parietal lobe sulci, and engorged choroid within the left lateral ventricle. The presumed supply by the middle cerebral artery and drainage via the basal vein of Rosenthal were again demonstrated on MRI, and no hemorrhage or infarction was identified. The brain parenchyma in the region of the vascular malformation showed no abnormal signal intensity. The diagnosis of DPCA was confirmed on a catheter digital subtraction diagnostic cerebral angiogram (Figure 2), which showed early venous drainage, no well-circumscribed focal nidus, involvement of multiple arteries of normal to mildly prominent size rather than a dominant feeder, and puddling of contrast persisting into late arterial and early venous phases.

**Figure 3:**
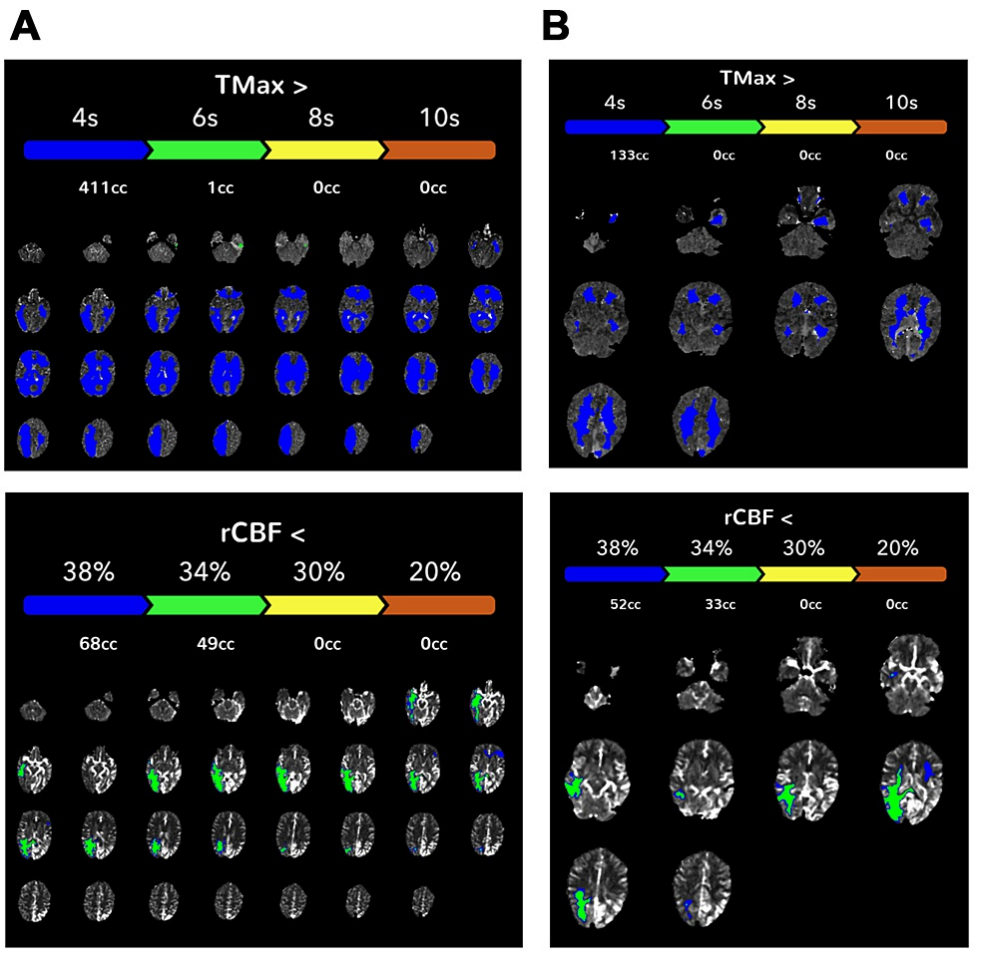
CT perfusion imaging at initial imaging. (A) Pre-acetazolamide studies reveal diffusely increased time-to-maximum (Tmax; >4 seconds) in bilateral cerebral hemispheres and decreased regional cerebral blood flow (rCBF) in the left parietal and temporal lobes. (B) Upon acetazolamide administration, there was an improvement in the hemodynamics metrics, with a decreased extent of prolonged Tmax.

Owing to the patient's persistent aphasia, we were concerned that the symptoms were attributable to a steal phenomenon. CT perfusion imaging revealed increased time-to-maximum (Tmax), which reflects the time delay between contrast bolus arriving in the proximal vessel arterial circulation and the brain parenchyma in bilateral cerebral hemispheres and increased regional cerebral blood flow within the DPCA (Figure 3). This suggested that there was increased blood flow within the DPCA and relative hypoperfusion in the surrounding area. An acetazolamide challenge, which vasodilates cerebral vasculature, was performed. After acetazolamide administration, there was a decrease in the extent of parenchymal involvement with a decreased extent of prolonged Tmax and decreased relative cerebral blood flow. This suggests increased parenchymal perfusion with vasodilation and decreased vascular steal.

The patient was started on a daily dose of 50 mg of cilostazol with the aim of inducing a vasodilatory response within the previously hypoperfused brain parenchyma. Within three days of treatment, the patient showed significant improvement in the ability to name objects, state their function, speak longer phrases, and comprehend speech. On day nine of hospitalization, the patient was discharged to inpatient rehabilitation, where he showed continued signs of improvement.

**Figure 4:**
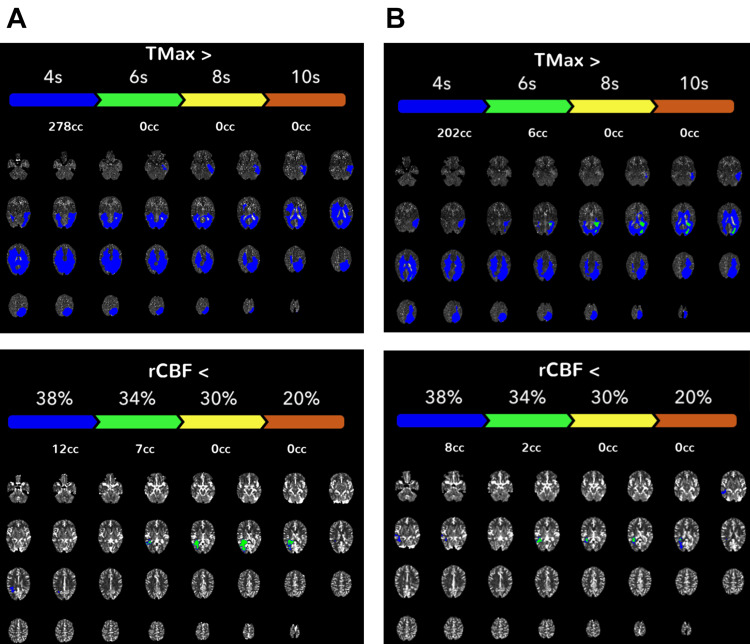
CT perfusion studies at six-month follow-up demonstrating improved hemodynamics compared with initial presentation A) Baseline time-to-maximum (Tmax) and regional cerebral blood flow (rCBF) pre-acetazolamide study in the left parietal temporal lobe. B) Tmax and rCBF after acetazolamide administration.

The patient returned to the clinic one month later and reported continued improvement in his aphasia with speech therapy. CTA showed an interval decrease in prominence of the vascularity in the left parietal, temporal, and occipital lobes. A follow-up CT perfusion study performed at six months showed a decreased extent of prolonged Tmax and relative cerebral blood flow (rCBF) before acetazolamide administration compared with the initial presentation (Figure 4). At the time of initial presentation, the pre-acetazolamide studies revealed diffusely increased Tmax (411 mL at Tmax >4 sec) and decreased rCBF (68 mL at rCBF <38%). After acetazolamide administration, there was a decreased extent of prolonged Tmax (133 mL at Tmax >4 sec) (Figure 3). At the six-month follow-up, the pre-acetazolamide Tmax >4 sec was 278 mL, and rCBF<38% was 12 mL (Figure 4). This decreased Tmax in the pre-acetazolamide study suggested that the patient's relative hypoperfusion at baseline improved with cilostazol treatment. However, it is important to note that cilostazol was administered with the aim of increasing perfusion to surrounding brain parenchyma to alleviate the patient's symptoms rather than to cure the underlying vascular pathology.

## Discussion

Although generally classified with AVMs, DPCA distinctly lacks a dominant arterial feeder and true nidus and frequently demonstrates proximal stenosis of feeding arteries, a transdural arterial supply of both normal and pathologic sites, capillary angioectasia, and moderately enlarged veins [1, 6]. The pathogenesis of DPCA remains unclear, but because of the similarities with moyamoya disease, in which internal carotid artery stenosis and resultant ischemia lead to increased angiogenesis, it has been hypothesized that a similar mechanism is responsible for the pathology seen with DPCA [2, 3, 7]. Specifically, the diffuse angiogenesis seen in DPCA is thought to be a response to cortical ischemia due to arteriovenous shunting [1, 2].

Steal phenomena in the setting of vascular malformations such as AVMs are commonly reported [8]. It is thought that the low resistance within the nidus of an AVM redirects blood flow toward it, causing relative hypoperfusion in the surrounding brain tissue [9, 10]. This relative hypoperfusion can manifest in clinical symptoms even without rupture [11]. Similarly, DPCAs such as those described here also form a vascular network with low resistance that increases perfusion within the DPCA but leads to hypoperfusion in the surrounding tissue. Our patient's aphasic symptoms were likely due to the relative hypoperfusion of the surrounding Wernicke's and Broca's areas. 

The presence of normal brain tissue entrapped within the malformation provides a clinical challenge in treating DPCA. The risk of permanent neurological damage is significant with intervention, whereas the risk of catastrophic hemorrhage is relatively low. Thus, most cases of DPCA are managed conservatively with antiepileptics or acetazolamide [2, 3, 12]. For patients who present with uncontrollable headaches or seizures, arterial embolization of noneloquent areas or revascularization with calvarial burr holes may be considered [1-3]. With the proposed etiology of DPCA, revascularization may be a logical treatment choice in select cases to address abnormal arteriovenous shunting and hypoperfusion-induced angiogenesis.

Our patient presented with aphasia, likely due to hypoperfusion of Wernicke's area (receptive speech) and Broca's area (speech production). The observed clinical improvements after administration of cilostazol, which has been shown to have antiplatelet, antiproliferative, vasodilatory, and ischemic-reperfusion protective properties [13], provide some evidence of the efficacy of its use for DPCA. The proposed mechanism of improvement is increased perfusion at sites adjacent to the DPCA, allowing for increased blood flow and correction of malformation-induced hypoperfusion. This finding further supports the proposed pathogenesis of DPCA because shunting in adjacent tissues would indeed lead to symptom improvement. Given that this patient improved with the administration of acetazolamide, the risk-benefit profile of surgical vs. medical management strongly favored medical management, and he was able to avoid surgical intervention.

It is important to note that no causal relationship between cilostazol administration and symptom resolution of DPCA can be drawn from the presented case. Rather, this case study demonstrates a noninterventional treatment option with a vasodilatory agent and functional rehabilitation. Further studies are needed to evaluate the true efficacy of cilostazol for the treatment of steal phenomena secondary to DPCA; however, because of the relative rarity of the pathology, these studies are difficult to perform.

## Conclusions

Surgical treatment of DPCA is difficult because normal brain tissue is intermingled within the malformation, so medical management should be given significant consideration and weighed against potential neurological deficits incurred with aggressive operative intervention. Our use of cilostazol for the management of the steal phenomenon secondary to DPCA suggests its potential as an efficacious medical treatment.
